# The intersections of migration, app-based gig work, and career development: implications for career practice and research

**DOI:** 10.1007/s10775-022-09556-w

**Published:** 2022-06-21

**Authors:** Peyman Abkhezr, Mary McMahon

**Affiliations:** 1 School of Applied Psychology, Griffith University, Parklands Drive, Southport, Gold Coast, QLD 4215 Australia; 2 School of Education, The University of Queensland, Brisbane, Australia

**Keywords:** Gig economy, Immigration, Career development

## Abstract

The incidence of app-based gig work is expanding rapidly in developed global north countries. Many app-based gig workers are migrants from developing global south countries searching for a better life in their resettlement countries. App-based gig work, however, is insecure, irregular and potentially precarious. Access to decent work is vital for migrants’ integration after resettlement and also their career development. In the context of the decent work agenda, this article explores the intersections of migration, app-based gig work, and southern migrants’ career development in the global north and considers the implications for career practice and research.

## Résumé

***Las intersecciones de la migración, los conciertos basados en apps y el desarrollo de la carrera*: implicaicones para la práctica de la carrera y la investigación.** La incidencia de el trabajo musical basado en apps se está expandiendo rápidamente en los países del norte global desarrollado. Muchos migrantes músicos que trabajan a partir de las apps desde países del sur global en dsarrollo, buscando una vida mejor en los países de reasentamiento. El trabajo musical basado en apps es inseguro, irregular y potencialmente precario. El acceso al. Trabajo digno es vital para la integración de los migrantes tras su reasentameinteo y también para su desarrrollo de la carrera. En el contexto de la agenda sobre el trabajo digno, este artículo explora las intersecciones del trabajo musical basado en apps y el desarrollo de la carrera de los migrantes del sur en el norte global y considera las implicacions para la práctica de la carrera y la investigación.

## Abstract (de)

**Die Überschneidungen von Migration, App-basierter Gig-Arbeit und Karriereentwicklung: Implikationen für die berufliche Praxis und Forschung.** Die Verbreitung von App-basierter Gig-Arbeit (eng. Gigwork) nimmt in den entwickelten Ländern des globalen Nordens rasch zu. Viele App-basierte Gigarbeiter sind Migranten aus Entwicklungsländern des globalen Südens auf der Suche nach einem besseren Leben in ihren Einwanderungsländern. App-basierte Gig-Arbeit ist jedoch unsicher, irregulär und potenziell prekär. Der Zugang zu menschenwürdiger Arbeit ist für die Integration von Migranten nach der Neuansiedlung und auch für ihre berufliche Entwicklung von entscheidender Bedeutung. Im Kontext der Agenda für menschenwürdige Arbeit untersucht dieser Artikel die Überschneidungen von Migration, App-basierter Gig-Arbeit und der beruflichen Entwicklung von Migranten aus dem Süden im globalen Norden und betrachtet die Auswirkungen auf die berufliche Praxis und Forschung.

## Resumen

**Les intersections entre la migration, le travail de plateforme basé sur des applications et le développement de carrière: Implications pour la pratique et la recherche en matière de carrière.** L'incidence des emplois de plateforme basés sur des applications se développe rapidement dans les pays développés du Nord. De nombreux travailleurs de ce type sont des migrants originaires de pays en développement du Sud à la recherche d'une vie meilleure dans leur pays de réinstallation. Cependant, le travail de plateforme basé sur des applications est peu sûr, irrégulier et potentiellement précaire. L'accès à un travail décent est vital pour l'intégration des migrants après leur réinstallation et pour le développement de leur carrière. Dans le contexte de l'agenda pour le travail décent, cet article explore les intersections entre la migration, le travail de plateforme basé sur les applications et l'évolution de carrière des migrants du Sud dans le Nord et examine les implications pour la pratique et la recherche en matière de carrière.

## Introduction

The twenty-first century has seen rapid expansion of the gig economy. While gig work has a broad scope and includes irregular and non-traditional work such as “on-demand work”, “crowd work” (De Stefano, 2016, p. 471), and “internet mediated freelance work” (Oyer, 2020, p. 3), this article focuses on “app-based gig work” (Oyer, 2020, p. 3). Examples of common app-based gig work include rideshare driving for companies such as Uber or DiDi, or food delivery for companies such as Uber Eats, Deliveroo or GrubHub. Electronically mediated employment arrangements govern app-based gig work.

Growing numbers of people are engaged in app-based gig work, many of whom in developed western countries such as Australia, the United States of America, Canada, and the United Kingdom, sometimes referred to as the global north, are migrants from developing nations, sometimes referred to as the global south^1^ (Pautuzzi & Benton, 2019; van Doorn et al., 2020; Victorian Government, 2020). Gig workers are regarded as self-employed and thus, are responsible for organising and planning their work which offers little, if any, of the security and benefits provided through other forms of traditional work found in developed countries (Kost et al., 2020).

Work is important for the integration of migrants into their countries of resettlement as well as their physical and psychological well-being (Abkhezr et al., 2015; Magnano et al., 2021; Swanson, 2012). For migrants, app-based gig work could be a double-edged sword (Bandeira, 2019; Pautuzzi, & Benton, 2019); while it could be a pathway to self-sufficiency and financial independence, it can also result in irregular salary, insecure work, a loss of skills, and limited career development (Bajwa et al., 2018; De Stefano, 2016; Prassl, 2018). Migrants, particularly those from the global south, may find themselves in the situation of having moved from precarious work situations in their home countries to being employed in precarious work in the app-based gig economy of their global north country of resettlement.

The expansion of app-based gig work is occurring at a time when more attention is being paid to trying to improve conditions for workers through the decent work agenda by countries around the world and by the career development profession (e.g. Blustein et al., 2019). The decent work agenda was proposed by the International Labour Organization (ILO) and is conceptualised through four main attributes, specifically: promotion of stable employment by sustainable institutional and economic contexts, protection for workers that includes social security and labour protection, moving towards a more dignified and just workplace, and finally, promotion of social dialogue across stakeholders to consistently achieve such attributes (ILO, 2008).

Gig work in the post-migration context might be reproductive of similar working conditions that migrants left behind in their global south countries of origin. This means that many gig workers do not have access to decent work. Since decent work provides conditions for workers that are largely absent from app-based gig work, the situation warrants attention by vocational psychology and career development researchers and practitioners. Continuous access to decent work is linked to sustainable career development (Urbanaviciute et al., 2019) which makes a positive contribution to workers’ health, happiness, and productivity (De Vos et al., 2020). Sustainable career development enables workers to benefit from dynamic employment arrangements that provide continuous learning, periodic renewal, some degree of security and stability, and a harmonious fit with their skills, interests, and values (De Vos et al., 2020). Consideration of the complex and multiple challenging experiences faced by migrants when they begin their post-migration career journey is essential in the context of the decent work agenda.

This article explores the intersections of migration, app-based gig work, and career development of southern migrants in the global north and considers the implications for career practice and research. A more detailed focus on the intersections of app-based gig work and career development after migration could not only enhance the livelihood of future migrants but could also reciprocally improve the intended outcomes of global north countries’ migration programs. Beginning with an overview of gig work, the article then considers migrants from the global south and the intersection of migration and app-based gig work in the context of the decent work agenda. The article concludes with suggestions for career practitioners and researchers.

## Gig work

The size of the gig economy is hard to measure, given that such data is not easily accessible because it is often controlled by platforms, and because many gig workers engage with gigs on a part-time or casual basis, or do not consider it as their primary work (De Stefano, 2016; Oyer, 2020). It is clear that the gig economy is expanding rapidly (ILO, 2020). For example, in 2019, the sixth annual study by the Freelance Union revealed that in the United States, over 57 million Americans freelanced among whom nearly 77% found their work online which partly explains the size of app-based gig work in the US (Upwork, 2019). The ride sharing industry is the largest provider of app-based gig work and it was estimated that by the end of 2019, Uber had more than 5 million drivers globally (Uber, 2020) and other platforms such as Didi or Lyft had similar numbers across different countries.

Other global north countries have also seen a rapid growth of their gig economy. A recent Australian study reported that over two thirds of the country’s population now routinely rely on app-based gig work platforms for purchasing goods and accessing services (McDonald et al., 2019). In Australia, the precise number of people who rely on the gig economy for their income is not available. However, the trends show a great increase in gig economy's size over the past few years, while migrant workers are found to be 1.5 times more likely to engage in gig work (Victorian Government, 2020).

The gig economy is transforming the lives of many people in different ways and contributing to rapid reconfigurations of the nature of work and work relations around the globe. However, the gig economy is still mostly conceived as a separate silo in the economy (De Stefano, 2016). Limited, or non-existent relevant policy or hastened legislative responses leaves gig workers with limited protection and support against unfair treatment and abuse by platforms that are not considered as employers (van Doorn et al., 2020). Some researchers have argued that until app-based gig work is legally recognised as work, gig workers will be mostly viewed as extensions of platforms, apps, and IT-devices (e.g. De Stefano, 2016), and therefore, remain in a vulnerable position. Despite some recent legislative responses that seek to recognise gig workers as formal employees (O’Connor, 2021), labour market policies that inform workers’ rights in the global north countries have not yet introduced and embraced policy that clarifies all aspects of app-based gig work. Consequently, dehumanisation of app-based gig workers prevails (He et al., 2021).

The recent challenges of the global pandemic have made the vulnerabilities faced by app-based gig workers even more apparent (Kantamneni, 2020). Globally, app-based gig workers have been impacted more than other groups of workers (Spurk & Straub, 2020) because many did not qualify to receive government support for reasons such as temporary work visas or not being recognised as unemployed (Kassel, 2020). While the livelihood and well-being of many gig workers remain at risk, app-based gig work and its complexities affect certain groups of people more than others.

For people with migration backgrounds, a stable and secure career pathway is linked with physical and psychological well-being, as well as cohesion in societies (Magnano et al., 2021; Swanson, 2012). Similarly, the career development of people with migration and refugee backgrounds is strongly linked to their integration and well-being (Abkhezr et al., 2015; Wehrle et al., 2018). Across many countries, a group that has been recognised repeatedly as vulnerable is those with migration backgrounds who have recently transitioned into global north countries from the global south countries (Biagi et al., 2018).

## Southern migrants in the global north

The term global north refers to most of the Organisation for Economic Cooperation and Development (OECD) countries and is an alternative designation for economically developed countries such as Australia, Canada, the United States of America, United Kingdom or New Zealand, regardless of their geographic location in the world. The world of work in many of the developing countries of the global south, where many migrants, and people with refugee and asylum-seeking backgrounds come from, is different from the rapidly shifting world of work of the global north. Despite the influences of globalisation and advances in technology on labour markets in global south countries, the nature of work in most of these countries remains bound to agrarian and industrial economies, as opposed to the service economy that forms a major part of the global north economy. In addition, the economic structure of the global south is characterised by “an abundance of labour and a scarcity of capital” (Cazes & Verick, 2013, p. 8) and the majority of workers in the global south work in the informal economy (ILO, 2019). Compared to their counterparts in the global north, workers in the global south are often in much more challenging competition for decent work and therefore, are more prone to mass unemployment, poor working conditions, wage gaps, discrimination, gender disparities, and even child labour (Biagi et al., 2018; van Doorn et al., 2020).

Many of the global north countries pursue migration programs such as “skilled migration”, “family migration”, as well as “humanitarian migration” programs (Australian Government, 2020). These programs are designed to sustain global north countries’ population growth rates and tackle challenges such as ageing populations, to foster economic growth in the contexts of an abundance of capital, and a scarcity of labour (Cazes & Verick, 2013). Migrants from global south countries constitute nearly 75% of all global migrant population. In 2019, out of all global migration, approximately 35% was from the global south to the global north, while nearly 40% migrated within the global south (United Nations, 2019). For example, Australia’s permanent migration program in 2019–2020 offered more than 140,000 places for various groups of migrants (Australian Government, 2020). Over 110,000 of these migrants (approximately 78%) were from countries that are considered as the global south (Australian Government, 2020). Since 2010, each year nearly 200,000 migrants have called Australia home, and by the end of 2019 over 60% of Australia’s approximately 1.5% population growth rate was due to net overseas migration (Australian Bureau of Statistics, 2020). In Canada (with almost a similar population growth rate), a similar trend has been observed over the past 10 years. On average, each year more than 270,000 migrants (Government of Canada, 2019) have contributed to Canada’s population growth rate indicating an even higher dependency on net overseas migration. Another example highlighting the importance of migration for the global north countries is the case of Japan, which had historically resisted the idea of permanent settlement of migrants. In 2019, however, Japan announced its intention to attract nearly 350,000 skilled workers to fill labour market gaps and also tackle the complexities of an ageing population (Ministry of Foreign Affairs of Japan, 2019). These trends are evident in many global north countries and reflect the inevitability of the continuation of southern migrants’ transition into the world of work of the global north with hopes of decent work.

## A southern perspective on migration

Migration may be considered from the perspective of the global north’s countries of resettlement as reflected in the previous section, or from the perspective of the global south. However, this article adopts a “southern perspective on migration” (Munck, 2008, p. 1227) that challenges the predominant global north discourse which views migration primarily as a beneficial process for the migrants, their families, and even their countries of origin. The current predominant discourse on migration (i.e. the northern perspective) has a specific focus on migrants in the global north who originate from the global south and amplifies two narratives: international migration as a threat and migration as an irregular act (Awad & Natarajan, 2018). By way of difference, the southern perspective considers “migration as a human right” (Oberman, 2016, p. 32). In this article, we consider a southern perspective on migration as a framework to enrich the exploration of the intersections of migration, gig work and career development. Adopting the southern perspective on migration as a human right aligns with the social justice foundations of vocational psychology and career development that also advocate for “decent work as a human right” (Blustein et al., 2019, p. 4). Simultaneous consideration of both migration and decent work as human rights means that the southern perspective potentially offers a new vantage point when it comes to other domains such as forced migration.

The southern perspective on migration aligns itself with the need to “broaden the notion of forced migration” (Wise, 2018, p. 163) as many factors such as the volatile economic and political contexts of many countries within the global south or in some cases, anthropogenic climate change and even the implications of the recent COVID-19 pandemic, further contribute to ongoing instability in the global south (Akkermans et al., 2020). As the southern perspective broadens the concept of forced migration, a need to revise what we have so far considered from a northern perspective as voluntary migration emerges. Structural injustices and the dynamics of uneven development in the twenty-first century continue to inflate the proportion of marginalised and disadvantaged populations in the global south. As such, according to the southern re-conceptualisation of forced migration, a large number of those marginalised and disadvantaged populations who will potentially need to immigrate due to dispossession, exclusion, and unemployment, including more than 600 million international and internal economic migrants could be considered as forced migrants (Wise, 2018). The systems that police incoming migration in the global north, through which the healthy, active, skilled, talented, and resourceful migrants are filtered, is often underestimated. Under the capitalist restructuring of the world while forced migration towards developed countries continues, migration simultaneously contributes to economic growth of the global north and further degrading of socioeconomic conditions in the global south by the “deepening of precarisation” and “cheapening of the workforce” in southern countries (Munck, 2008; Peticca-Harris et al., 2020; Wise & Covarrubias, 2009, p. 99). Changes at a global level impact southern migrants and sometimes follow them into more vulnerable positions after migration. Post-migration is filled with experiences of downward career mobility, lack of access to decent work, and career depreciation for many migrants (Rajendran et al., 2020). Over the past few years, app-based gig work has undoubtedly emerged as a new major form of work in the post-migration context. However, as previously stated, it acts as a double-edged sword for many migrants (Bandeira, 2019).

## Gig work and migrants

App-based gig labour in many of the global north countries that have a substantial migration program is predominantly migrant labour (Pautuzzi & Benton, 2019; van Doorn et al., 2020; Victorian Government, 2020). Two possible factors might explain the relatively higher rates of migrants’ engagement with app-based gig work as opposed to non-migrant populations of the global north.

The first factor relates to the complex, systemic, and structural constraints and challenges that migrants face with their career development after migration. Many migrant groups, including people with refugee backgrounds, humanitarian migrants or those categorised as ‘skilled migrants’ (under the northern countries’ immigration systems), who transition from the global south to the global north, experience numerous challenges with their career development. These include but are not limited to lack of familiarity with the new country’s recruitment and employment markets, dis-recognition of their overseas qualifications, complexities, and long delays for assessment of their overseas qualifications, language barriers, systemic discrimination, and lack of access to relevant career resources and support (Abkhezr et al., 2015, 2018; McMahon et al., 2019; van den Broek & Groutsis, 2020; Zacher, 2019). Research from several disciplines such as social work (e.g. Saldanha, 2020; Zaviršek, 2017), public and industrial relations and policy (e.g. Australian Government, 2019; Goods et al., 2019; Biagi et al., 2018; Victorian Government, 2020), business and economics (e.g. Clark et al., 2019; Faaliyat et al., 2021), and organisational and vocational psychology (e.g. Arthur & Nunes, 2014; Blustein et al., 2019) points to the challenging task of adjusting to the rapidly shifting and different nature of twenty-first century work in the global north for those who were raised and educated in the global south and its often different world of work. Consequently, migrant workers are in a vulnerable position due to the challenging task of navigating a complex and unfamiliar system to secure and maintain safe, steady and sustainable employment after migration (van Doorn et al., 2020; Victorian Government, 2020).

The second related factor concerns the quick pathway to self-sufficiency and financial independence that app-based gig work appears to offer (Pautuzzi & Benton, 2019). The process of recruiting workers is usually quick for the platform companies that offer app-based gig work because they are not considered as employers, have no employment contracts and almost no commitment is needed from them. Platform companies market their work as a flexible work arrangement that offers workers high levels of autonomy and independence. Considering the challenges that many migrants might face with securing employment and their career development after migration, such attractive arrangements make it relatively easy for them to start their engagement with app-based gig work in order to maintain dignity and independence. However, for many migrants who have previous qualifications and work experiences, app-based gig work engagements pose a range of integration and career development risks. These include, but are not limited to, unstable income, insecure work, underemployment, decreased well-being, social isolation, skills depreciation and loss, and finally, highly disrupted career development (Bajwa et al., 2018; De Stefano, 2016; Prassl, 2018; Victorian Government, 2020).

The post-migration context could potentially position southern migrants in situations where oppressive forces adversely impact their career development and pose unexpected and unanticipated challenges when advancing their career plans (Blustein et al., 2019). Low wage, informal and precarious employment, underemployment, and sub-optimal work conditions that generally translate into a lack of decent work for migrant populations are increasing in the global north, and with it the proportion of the working poor (ILO, 2019). Future cohorts of southern migrants could be at an even greater risk of having to experience lack of access to decent work, and consequently make app-based gig work engagements inevitable. In addition, migration programs often focus more on attracting younger migrants, yet their future employment prospects could be threatened if a healthy and sustainable sense of career identity is not established (Wehrle et al., 2018). Hasty career and employment related decisions with lifetime implications, fuelled by the “traditional capitalist narrative” of working hard and achieving a dream (Peticca-Harris et al., 2020, p. 36), might further stretch the gap between migrants’ sustainable career development and their access to decent work.

## Career research and a decent work agenda

The importance and desirability of decent work as a human right and as an “antidote to precarious work” (Blustein et al., 2016, p. 4) has gained increasing attention. Recent publications have acknowledged the struggles that impact the career development of many people across the globe, creating barriers for a decent work agenda. For example, a special issue of *Frontiers in Psychology* explored the challenges of expanding the decent work agenda (see Blustein et al., 2016), and a special issue of the *South African Journal of Education* explored the possibility of a career counselling renewal to promote the facilitation of sustainable decent work (see Maree, 2020). In 2020, a special issue of the *Journal of Vocational Behavior* specifically focused on “a cross-cultural exploration of decent work” (see Duffy et al., 2020) and the Decent Work Scale (Duffy et al., 2017). Other theoretical and conceptual publications have addressed a wide range of perspectives that inform the decent work agenda and challenges that threaten sustainable decent work for all people across the globe (e.g. Blustein et al., 2019; Hirschi, 2018; Urbanaviciute, et al., 2019).

The need for a focus on a decent work agenda in the vocational psychology and career development fields is somewhat informed by the historical social justice roots of our fields. For example, the career needs of the migrants were a focus of the work of career pioneers and social activists such as Frank Parsons in the United States (1909), Etta St. John Wileman in Canada (Van Norman et al., 2014), and Carolyn Chisholm in Australia (Arthur & McMahon, 2019). However, “experience-near” (Geertz, 1983, p. 57) stories of people who struggle with their career development and are engaged in recent forms of precarious work, such as those engaged with app-based gig work, remain unexplored in career development research. The limited research on app-based gig work conducted in other fields, has so far revealed a diverse range of often contradictory experiences, as various groups of gig workers (depending on context, demographics or gig types) emphasise advantages or disadvantages of their work (Bandeira, 2019; Peticca-Harris et al., 2020). Such research has rarely focused on career development of southern migrants in the global north.

To explore the degree by which app-based gig work has been researched from a career development and vocational psychology perspective the terms ‘gig work’ and ‘gig economy’ were searched within nine career psychology journals (*Career Development International, Career Development Quarterly, Journal of Vocational Behavior, Journal of Career Assessment, Journal of Career Development, International Journal for Educational and Vocational Guidance, British Journal of Guidance & Counselling, Australian Journal of Career Development, and The Canadian Journal of Career Development*). This search revealed a total of 12 articles published up until November 2020 that focused on gig work. Only one article among the 12 explored gig work engagements and experiences with participants (Beigi et al., 2020) and another focused on the development and validation of Job Precariousness Scale (Creed et al., 2020). The other 10 publications were theoretical and conceptual.

In addition to theorising and conceptualisation on gig work, to progress vocational psychology and career development’s contributions to a decent work agenda, the intersections of career development, migration and gig work warrant further research as well as a consideration of how relevant career support could be provided for migrant groups, especially those from the global south. In considering practice and research implications of such intersections, we encourage readers to adopt the “southern perspective on migration” (Munck, 2008; p. 1227) which encompasses elements of: (1) migration and decent work as human right, (2) a revised conceptualisation of forced and voluntary migration and their overlapping boundaries, and (3) migration as a contributing factor to economic growth of the global north countries.

## Implications for career practice

Based on our southern perspective on migration, this section first considers how dominant career discourse practices marginalise possibilities for career practice, and then considers the promises and complexities of constructivist and narrative career practices when working with migrant gig worker clients. Practical suggestions based on narrative practices are then offered.

### Dominant career discourse practices and the marginalisation of possibilities

Dominant career discourse practices “psychologise and individualise vocational choice” (Richardson, 2012, p. 94) and promote a neoliberal approach to career development in which everyone presumably benefits from the same degree of career volition. Such a discourse runs the risk of marginalising a variety of alternative possibilities and solutions, as well as rendering invisible an array of challenges that certain clients face in their lives. There is little to guide career practitioners about how their work with migrants employed in the gig economy might inadvertently be reproductive of unhelpful career discourses.

Career discourse practices, like other disciplinary practices within any society, constitute a power/knowledge regime on these types of work (Foucault, 1980). For example, assumptions on the part of some career practitioners about gig work as a precarious form of work may not be reflected in the local conceptualisations of gig workers themselves. For some workers, the flexibility and autonomy that gig work offers is vital, while for others it may be viewed as complementary income or even an opportunity to socialise (Beigi et al., 2020; Peticca-Harris et al., 2020). Thus, a more local conceptualisation of gig work by gig workers themselves may reflect simultaneous acknowledgement and rejection of its precariousness (Peticca-Harris et al., 2020). How career practitioners view and approach their client’s gig work engagements could be imposed on clients through the power relations that exist in their work with clients. Highlighting only the precarious nature of gig work in conversations with clients, or simply accepting and moving on with the flexibility narrative, could both be reproductive of dominant discourses and marginalise clients’ local knowledges and preferences that have a potential to enrich career conversations, and ultimately career counselling outcomes.

For instance, dominant career discourse practices have led to marginalisation of “care work” that can be seen as “social reproduction” (Richardson, 2012, p. 95). As the majority of southern migrants are from collectivist cultures in which family and relational dimensions of identity have a more central role (Kagitcibasi, 2005), a reconsideration of the nature and meaning of work might be helpful and could potentially lead to a more curious approach to the interplay between gig work and care work. After migration, people lose many of their social and relational resources, and their nuclear family begins to play an even more central role in sustaining their well-being and sense of identity. Considering the role of app-based gig work in contexts where “social reproduction” and “care work” might have an equal weight with “economic production” and “paid work” (Richardson, 2012, p. 95) may reveal the importance of attending to local and particular narratives when working with southern migrants (Sultana, 2021). Conversations related to the intersections of app-based gig work, family, relational and social aspects of life in the post-migration context could open up possibilities of new stories for both clients as well as their career counsellors.

Career practitioners who adopt a southern perspective on migration are context-sensitive and apply an anthropological lens to exploring and understanding the diversity of migrant clients’ experiences and worldviews, and also the challenges and possibilities that gig work might offer them. This contextual and curious approach could lead to context-responsive ways of working with migrants that essentially align with constructivist career practice.

### Constructivist career practice: promises and complexities for working with gig worker clients

The constructivist practice of narrative career counselling has been proposed as a potentially promising approach when working with people from refugee or migration backgrounds because it prioritises subjectivities, is context-sensitive, and accommodates cultural and contextual diversity (Abkhezr & McMahon, 2017; Arthur, 2017; Magnano et al., 2021; Watson, 2017). Narrative career counselling highlights the dialogical and storytelling dimensions of working with people (McMahon & Watson, 2013).

As we are embedded in a social and relational context, in our day-to-day socialisations we are exposed to various stories and dialogical spaces, and the socially constructed class or value of a given work area. Even career counselling itself is a social and dialogical space. As career practitioners assist people with making informed career choices, plans, and decisions they engage in acts of storytelling and extend invitations for constructing new stories of possibilities. The newly constructed stories open space for new interpretations and values to be constructed by the client. However, as these conversations are subject to various operations of power in the counselling space, they could become reproductive of traditional and dominant career discourse practices that value certain types of work and devalue other types. One aspect of such complexities is related to the existence of uncertain, ambiguous, and often subjective conceptualisations of work and career between the practitioner and the client (one seeing it more from the neoliberal perspective and believing in the grand narrative of ‘*everyone can achieve a career with hard work’*, and the other not really following or agreeing with this). Although with good intentions, such potential discursive disciplinary influences might not always benefit clients. For example, a client might get the impression that the career practitioner is not approving of their gig work and devaluing it for their career development. Complexities related to the application of narrative career counselling (that might at least be the most context-sensitive way of working with southern migrants) and lack of research, makes it more challenging to determine in what ways the dominant discourses in our field, aligned with a neoliberal conceptualisation of gig work, are constituting a power/knowledge regime, and as a result influencing career conversations with southern migrants.

## Practical possibilities for narrative career practice with southern migrant gig workers

Providing a “voice friendly space” (Abkhezr et al., 2018, p. 28), sensitive to subjective experiences and conceptualisations of clients is a central component of narrative career practice and should be a priority. Engaging in conversations that do not position or conceptualise gig work engagements as secondary or non-career initiatives is another priority for narrative career practitioners. Alternatively, one possibility is the exploration of client’s preferred position on the role of app-based gig work, as an externalised entity (see externalising conversations in narrative therapy; e.g. White, 2007). Once *App-Based Gig Work* is externalised, its role, position and operations in the client’s life could be narrated and evaluated more carefully. In such conversations, gig work can be potentially characterised on a continuum, ranging from a problem to a short-term solution. Adopting an externalising approach to conversations around app-based gig work, could then facilitate further rich and thick conversational possibilities through “Statement of Position Maps” (White, 2007). In such conversations, client and counsellor together can revisit the location of these types of work on the continuum each time they meet or when new understandings, values or plans emerge in the client’s life. After a careful exploration of the operations of app-based gig work in a client’s life, and collaboratively evaluating any emerging post-migration values, plans, understandings, events or developments in relocating the position of app-based gig work on the continuum, a new exploratory stage of conversations might begin. This next exploration phase could focus on the experience of new developments, initiatives, events, or values that might have emerged and evolved as a result of the relocation of app-based gig work on the continuum.

## Implications for research

Research on the intersections of app-based gig work, migration and career development could consider the oppressive forces that surround people after migration in a complex world of work in the global north, and the multiplicity of various groups of people’s local and personal experiences with app-based gig work and how they impact career development. A number of propositions are outlined for research, with some of them relevant for researching career practice. By adopting a southern perspective on migration, these propositions prioritise social justice as one of the core values of career practice and research and intend to stimulate innovative research and practice.

### Heterogeneity of migrants engaging in app-based gig work

Southern migrants are a heterogenous group of people, particularly when their migration journey, education, work experiences, and career development are concerned. Migrants who engage in app-based gig work after migration might perceive their gig work experiences differently depending on their backgrounds and post-migration circumstances. Qualitative research inquiries could enhance understandings of migrants’ experience of app-based gig work by prioritising experience-near narratives that facilitate the disentangling of the intersections of gig work, migration, and career development in a more personally preferred way. In doing so, a more nuanced understanding of the cultural and contextual complexities that contribute to the career development of southern migrants in the age of gig work could be considered in research.

### Operations of culture in the intersections of gig work and migration

Career research needs to be culture sensitive when studying the intersections of gig work and migration. The diverse cultural backgrounds of migrants contribute to varied migration expectations, as well as a diverse range of meanings assigned to work and career development (Abkhezr et al., 2018; Di Fabio & Kenny, 2016). Sensitivity to culture when working with southern migrants means adopting “culturally appropriate research methodologies” (Flores et al., 2019) that require researchers to consider the potential challenges of participation for disadvantaged groups, such as low-income app-based gig workers, and carefully evaluate the usefulness and potential benefits of research participation when designing research.

### Possibilities for researching career practice with southern migrant gig workers

As little research has so far revealed the diversity of southern migrant gig workers’ lived experiences and how their gig work intersects with migration and career development, the role of career practice in responding to such intersections could be explored. Researching career practice could contribute to greater understanding of marginalised knowledge and promote the importance of practice-based evidence, that could bridge the gap between career practice and research.

Research could focus on questions such as how career practitioners position themselves in relation to southern migrants’ gig work; to what degree career practitioners perceive app-based gig work aligns with possibilities for sustainable career development of southern migrants; to what degree career practitioners adopt a southern perspective on migration when they work with such clients; and what cultural and contextual understandings inform career practitioners’ work with southern migrants. For example, narrative career counsellors prioritise cultural and contextual conversations in their practice (Reid & West, 2016), but lack of research on narrative career counselling requires future research to explore the type of conversations and dialogical spaces narrative career practitioners facilitate for southern migrant gig workers. Finding answers to such questions could stimulate the adoption of a southern perspective on migration in career research and practice consistent with the social justice roots of career development.

### Towards social mobility with responsible research

To ensure that social justice remains central to career research, it is also essential that southern migrant gig workers who are engaged in career research, are at the same time assisted with their social mobility and career development, and not viewed only as participants or research subjects. Participatory and voice-centred research, participatory action research, emancipatory research, and narrative inquiry are all forms of research that in different ways contest “the privileging and separation of expert knowledge” (Byrne et al., 2009, p. 67), and aim to give space to the marginalised voices of participants, enhance their sense of agency, improve their sense of community and belonging, and utilise their input to inform policy, practice, and future research. In various disciplines, these forms of research have also resulted in community development and grassroots action for different groups of disadvantaged populations such as people with refugee and asylum-seeking backgrounds (Duarte et al., 2018; Quinn, 2012) and migrant workers (Bhuyan et al., 2018). Finally, narrative research that is sensitive and responsive to the “ethics of care” (Clandinin, 2007, p. 30) could also cross boundaries with narrative interventions that aim to enhance clients’ reflectivity and sense of agency (Abkhezr et al., 2020).

## Conclusion

App-based gig work is becoming a common feature of labour markets in the countries of the global north. The app-based gig workforce includes many migrants, including those from the global south. To date, little is known about the intersection of migration, app-based gig work and career development. Career research that is culturally sensitive and allows the voices of migrant participants to be heard may provide useful insight into their reasons for and experiences of engaging in app-based gig work. Such research may also serve to challenge dominant discourses and inform the work of career practitioners.

## Footnotes

1 The terms global south and global north are used to describe the grouping of countries along socioeconomic and political characteristics, and do not refer to geographical location. For example, even though Australia and New Zealand are located in the Southern hemisphere, their western, wealthy socioeconomic status positions them as global north countries.
